# Phenotypic and Genetic Effects of Contrasting Ethanol Environments on Physiological and Developmental Traits in *Drosophila melanogaster*

**DOI:** 10.1371/journal.pone.0058920

**Published:** 2013-03-07

**Authors:** Luis E. Castañeda, Roberto F. Nespolo

**Affiliations:** 1 Departament de Genètica i de Microbiologia, Grup de Biologia Evolutiva (GBE), Universitat Autònoma de Barcelona, Bellaterra (Barcelona), Spain; 2 Instituto de Ciencias Ambientales y Evolutivas, Facultad de Ciencias, Universidad Austral de Chile, Valdivia, Chile; Fred Hutchinson Cancer Research Center, United States of America

## Abstract

A central problem in evolutionary physiology is to understand the relationship between energy metabolism and fitness-related traits. Most attempts to do so have been based on phenotypic correlations that are not informative for the evolutionary potential of natural populations. Here, we explored the effect of contrasting ethanol environments on physiological and developmental traits, their genetic (co)variances and genetic architecture in *Drosophila melanogaster*. Phenotypic and genetic parameters were estimated in two populations (San Fernando and Valdivia, Chile), using a half-sib family design where broods were split into ethanol-free and ethanol-supplemented conditions. Our findings show that metabolic rate, body mass and development times were sensitive (i.e., phenotypic plasticity) to ethanol conditions and dependent on population origin. Significant heritabilities were found for all traits, while significant genetic correlations were only found between larval and total development time and between development time and metabolic rate for flies of the San Fernando population developed in ethanol-free conditions. Posterior analyses indicated that the G matrices differed between ethanol conditions for the San Fernando population (mainly explained by differences in genetic (co)variances of developmental traits), whereas the Valdivia population exhibited similar G matrices between ethanol conditions. Our findings suggest that ethanol-free environment increases the energy available to reduce development time. Therefore, our results indicate that environmental ethanol could modify the process of energy allocation, which could have consequences on the evolutionary response of natural populations of *D. melanogaster*.

## Introduction

One of the most important research programs in evolutionary biology is the characterization of the genetic architecture of morphology, performance and fitness in natural populations [1]–[4]. The central formalization of this theoretical framework is the multivariate breeder's equation, which establishes that the mean phenotypic response vector is equal to the additive genetic (co)variances matrix (G matrix) multiplied by the vector of selection gradients (β) [4], [5]. However, an important assumption for the long-term validity of this model is that G matrix must remain stable through ecological time (e.g, less than hundred generations, [4], [6]). The selection-mutation balance would support this assumption because genetic variation is continuously eroded by directional or stabilizing selection, but is also increased by *de novo* variance due to mutation and recombination [3], [4], [7]. However, several reviews and meta-analyses have pointed out that natural selection varies from weak to very strong [8], [9], [10], suggesting that the mutation-selection balance could be an unwarranted assumption and hence the G matrix could vary considerably through ecological time and space.

Given that genetic architecture is the result of the multivariate distribution of breeding values, which in turn are the result of the expression of many genes in a given environmental condition, one of the main sources inducing genetic changes is environmental variability [11], [12]. Empirical evidence indicates that environmental factors such as temperature [13], metal concentration [14], predator presence [15] and food availability [16] act as modulators of genetic variances and covariances and, therefore of the G matrix structure. Similar to these environmental factors, ethanol varies at local and large spatial scales and is an important component of rotting fruit on which many species (e.g., nematodes and fruit flies) feed and breed during different stages of their life [17], [18]. Such is the importance of ethanol as a selective agent that evidence of clinal variation for increasing ethanol tolerance with latitude has been worldwide reported for *Drosophila melanogaster* [19], [20], [21]. *D. melanogaster* is known for breeding on and consuming rotten fruits with variable ethanol levels, ranging from beneficial to toxic levels [17], [22]. To cope with fluctuating dietary ethanol levels, *D. melanogaster* has several detoxifying enzymes such as alcohol and aldehyde dehydrogenases that degrade ethanol and aldehyde, respectively [23]. However, as with many other induced physiological defenses, ethanol detoxification can be associated with high energetic costs, which could have effects on fitness-related traits because a resource-allocation constraint [24]. This criterion is based on the fact that resources are limited and need to be allocated simultaneously to several functions, many of which have a direct impact on fitness [25], [26], [27]. Therefore, induction of physiological defenses should be negatively correlated with life-history traits, and hence trade-offs between physiological and fitness-related traits are predicted [28], [29]. Empirical evidence from *Drosophila* species supports this hypothesis: (*i*) flies increase their detoxifying enzyme activities when exposed to increasing ethanol levels [30]; (*ii*) flies exposed to ethanol vapor augment their metabolic rates [31]; and (*iii*) flies selected for high-ethanol tolerance increase their development times [24].

Several studies have highlighted the importance of the relationship between energy metabolism and fitness-related traits [32], [33], [34]. Therefore, it is plausible that ethanol consumption (via induced detoxification) could increase metabolic rates, reducing energy available for competing fitness-related traits. For instance, given that development time and body size are important contributors to fitness [26], [27], the genetic correlations between energy metabolism and these traits are especially informative because they represent a key link between physiological performance and fitness. Provided that enough genetic variation exists in either trait, we expected to find a positive genetic correlation between energy metabolism and development time and/or a negative genetic correlation between development time and body size for flies exposed to ethanol. This is because energy resources would be allocated to detoxification instead of growth (i.e., fitness costs would slow down development rate and produce small adults). In contrast, in favorable environments (e.g., ethanol-free conditions), ‘expensive’ traits such as detoxification systems would be ‘switched-off’ because their maintenance might entail fitness costs [11], [24]. In this case, a negative genetic correlation would be predicted between energy metabolism and development time because energy for detoxification now is available for other competing functions and thus individuals with higher metabolic rates would grow faster.

According to these predictions, our main goal was to test the effects of contrasting ethanol environments on physiological and developmental traits and their underlying genetic architecture in the fruit fly, *D. melanogaster*. To address this issue, phenotypic and genetic parameters were estimated in two populations, using a half-sib family design where broods were split into ethanol-free and ethanol-supplemented conditions. For each fly, we measured development time (larval, pupal and total development time), adult metabolic rate (i.e, routine metabolic rate) and adult body mass. Because metabolic rate is particularly difficult to measure in individual larvae, this trait was measured in adult flies, assuming that metabolic rates are correlated between both life stages (an assumption that is partly supported by Folguera et al. [35]). According to this experimental design and measured traits, (1) we evaluated the ethanol effects on phenotypic means of flies from two populations reared in contrasting ethanol conditions, (2) we estimated heritabilities and genetic correlations (as well as variance and covariance components) between traits, and (3) we tested if the resulting G matrices changed or remained stable between contrasting ethanol conditions.

## Materials and Methods

*D. melanogaster* has a global distribution and has no special conservation status in IUCN Red List, US Federal List and CITES [36]. Fly collection and experimental procedures were approved by the Comité Asesor de Bioética (Permit Number: 018/FONDECYT/1352) of the Fondo Nacional de Investigación Científica y Tecnológica (FONDECYT–Chile), and Comité de Bioética (Permit Number: 19/08) and Comité Institucional de Bioseguridad (Permit Number: 0080/08) of the Universidad Austral de Chile.

### Sampling and maintaining conditions

Flies were sampled from two populations in Chile: San Fernando (34°35′S–70°59′W) and Valdivia (39°48′S–73°14′W), using traps with banana and brewer's yeast. In both locations, female flies were sampled in apple orchards that received no insecticide applications. The owners of these private lands granted all necessary permits for the described field sample sites: Greenvic S.A. in San Fernando (Chile), and Universidad Austral de Chile in Valdivia (Chile).

Wild-caught females from each population were individually placed in glass vials containing ethanol-free medium and maintained at 20°C and 12:12 light/dark photoperiod. Eighty isofemale lines per population were dumped in an acrylic cage (27×21×6 cm^3^) to set up a large outbred population. At third generation, each population cage was split into three replicated lines maintained in individual acrylic cages as before. Prior to the experiments, replicated lines were maintained for four generations under ethanol-free conditions with at least 1000 breeding flies (see Santos et al. [37]).

### Experimental design

Seventy eggs were collected from each replicated line within population. These eggs were individually placed in 20-mL vials with ethanol-free medium. When flies emerged, they were sexed and one male (*sire*) was chosen randomly and transferred to a new vial where it was mated with four virgin females (*dams*) from the same replicated line. Three days after mating, each dam was individually placed in a vial and allowed to oviposit on ethanol-free medium, thus offspring developed under ethanol-free conditions. After 24 h, each dam was transferred to a new vial with a thin layer of ethanol-free medium and allowed to oviposit for a further 24 h. After this time, medium layers containing eggs were individually placed on fly's medium containing 7% ethanol, thus offspring developed under ethanol-supplemented conditions. The ethanol medium was prepared like the ethanol-free medium, but when temperature of the medium dropped to 45°C, 7 mL of absolute ethylic ethanol were added per each 93 mL of ethanol-free medium [24].

Our breeding design was established with 29 half-sib families for the San Fernando population (ten families from replicated cage 1, eight families from replicated cage 2, and eleven families from replicated cage 3) and 16 half-sib families for the Valdivia population (six families from replicated cage 1, five families from replicated cage 2, and five families from replicated cage 3). The lack of families from the Valdivia population was caused by a problem that occurred during maintenance and may have reduced the statistical power to detect significant genetic parameters for this population. Table S1 contains detailed information about sire, dam and sib sample sizes.

### Phenotypic traits

From vials of each experimental condition (i.e., ethanol-free and ethanol-supplemented condition), six larvae were randomly selected and individually transferred to vials containing medium with the same ethanol concentration as previously. After that, each larva with known oviposition date (see above) was checked every 24 h to record the pupae formation (e.g., yellow or brown pupae) to compute larval development time. Subsequently, each pupa was checked every 24 h to record adult eclosion and to determine the pupal development time. Total development time was also estimated for each individual accounting for oviposition date and eclosion date.

Twelve days after eclosion, flies were anesthetized using CO_2_, sexed, and their body mass (Mb) was recorded using a microbalance (MXA5, Radwag, Czech Republic) before metabolic rate measurements. A flow-through respirometric system was used to estimate the routine metabolic rate (RMR), which was measured as the CO_2_ produced in 10 minutes by a fly inside a 25-mL metabolic chamber at 20°C. In order to reduce the volume of the chamber, its inner space was filled with cotton to reduce its washout time [38]. For each metabolic record, seven metabolic chambers containing individual, free-moving flies (plus one chamber without a fly used as baseline) were connected to a gas flow multiplexer (Sable Systems International, NV, USA), which allowed the sequential measure of RMR of seven flies per record. Air was pumped into an acrylic column containing CO_2_ absorbent (Spherabsorb, Intersurgical, Berkshire, UK) at a continuous flow rate of 50 mL min^−1^, controlled by a mass flow controller (Sierra Instruments, CA, USA). Flies were maintained for 30 minutes in darkness within the metabolic chambers before the measurements of CO_2_ production in dark conditions to reduce the fly's movement. Experimental conditions were controlled with a PTC-1 temperature cabinet, connected with a PELT-5 temperature controller (Sable Systems International, NV, USA). These thirty minutes were enough for thermal acclimation and avoid CO_2_ anesthesia effects on metabolic measurements. CO_2_ produced by each fly was measured with a daily-calibrated Li-7000 CO_2_ analyzer with a resolution of 1 ppm (LI-COR Bioscience, NE, USA), which was connected to the multiplexer. The data output from the CO_2_ analyzer was recorded with the Expedata software (Sable Systems International, NV, USA). Each record includes the measurement of seven flies and the empty chamber, which was measured three times during the record (begin, middle and end of the record). Using the Expedata software, the three baseline points were used to establish a zero CO2 value for the entire record assuming a linear drift, and then the original data of ppm values of CO2 were transformed to metabolic rate (μl CO2 h-1) according to Artacho & Nespolo [34].

Given this respirometric design, approximately nine metabolic records (each one including seven flies) were performed per day, resulting in a total of 63 flies measured per day. Furthermore, we analyzed methodological effects on RMR caused by (*i*) chamber effects and (*ii*) hour at which metabolic measurement was performed (i.e., caused by any circadian effects on RMR).

### Statistics I: Phenotypic analyses

Normality and homoscedasticity were checked for each phenotypic variable. All variables were log_10_-transformed to fulfill parametric assumptions. Analyses of variance for larval, pupal and total development time, and adult body mass were performed using the following mixed linear model:
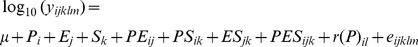
(1)where *µ* is the overall mean of the trait, *P_i_* is the fixed effect of population, *E_j_* is the fixed effect of ethanol treatment, *S_k_* is the fixed effect of sex, *r_l_* is the random effect of replicated lines nested within population, and *e_ijklm_* is the residual error term, with the corresponding interaction terms. RMR analyses were performed using the following linear model:
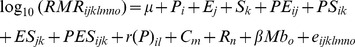
(2)where the same nomenclature applies from eqn. 2 with the addition of *Cm* being the fixed effect of metabolic chamber, Rn being the fixed effect of the hour at which the metabolic measurement was performed, and *βMbo* being the covariate effect accounted for adult body mass. Unequal N HSD tests were performed to evaluate a *posteriori* differences when effects were significant. Phenotypic analyses were performed using Statistica 6.0 software [39]. For all analyzed traits, a detailed statistical information is provided in Table S2.

### Statistics II: Quantitative genetic parameters

Since a half/full-sibs design was used, the pedigree structure of all measured individuals was known. Using this pedigree information, we estimated the additive genetic variance (*A*), common environmental variance (*C*), replicated line variance (*R*), non-common environmental variance or residual variance (*E*) and total phenotypic variance (*T*) for each population reared in each ethanol treatment. To estimate these variance components, we fit a generalized linear mixed model using a Monte Carlo-Markov Chain (MCMC) approach. These analyses were performed applying a modified version of the script provided in Wilson et al. [40] and the MCMCglmm package [41] available for the software R 2.13.2 [42]. Log_10_-transformed variables were used for the quantitative genetic analysis in order to avoid scale effects in variances. The following animal model was used to estimate the variance components of adult body mass, and larval, pupal and total development time:
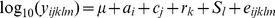
(3)where *μ* is the overall mean, *a_i_* is the random effect of breeding values of each individual, *c_j_* is the random effect of common-environment variation, *r_k_* is the random effect of population line from which each parent was sampled, *S_l_* is the fixed effect of sex, and *e_ijklm_* is the random residual error. For RMR analysis, the following animal model was used:
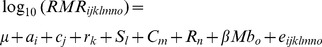
(4)where the same nomenclature applies from eqn. 3. *Cm* is the fixed effect of metabolic chamber, *Rn* is the fixed effect of the hour at which metabolic record was performed, and *βMbo* is the effect accounted for adult body mass as a covariate.

For running MCMC analyses, we specified prior values partitioning the total variance according to how many random effects were included in the animal model [43]. Therefore, the specified prior variance was equal to the *T* divided by four because our animal model included four random effects (*a_i_, c_j_, r_k_ and e_ijklm_*; see equations 3 and 4). Analyses were run for 1,000,000 iterations and the first 100,000 iterations were discarded (i.e., burn-in period) to ensure the parameter convergence at the maximum likelihood. The remaining iterations were sampled every 500 iterations to estimate variance components (i.e., *A*, *C*, *R* and *E*). Posterior distribution of additive genetic (co)variances were checked for all MCMC analyses through the evaluation of the autocorrelation within each chain, which was lower than 0.05 for all cases.

Using these variance components, we estimated heritabilities (i.e., *h* ^2^ = *A*/*T*) and their respective 95% confidence intervals. Genetic covariances and correlations (*r*_g_) were estimated in a similar fashion, but including pairs of traits in the models shown in equations 3 and 4. Genetic parameters (e.g., *h* ^2^ and *r*_g_) were considered statistically significant when their 95% confidence intervals did not overlap zero values, and comparing the deviance information criterion (DIC) between the complete model (*ACRE*) and the constrained model (*CRE*) (Tables S3–S6).

### Statistics III: G matrix comparisons

Matrix comparisons using Jackknife-MANOVA method [44] were performed between ethanol treatments by population because unbalanced family numbers between populations may not be informative about real differences (or similarities) of G matrices. This method firstly estimated an observed G matrix for each ethanol treatment within populations. Then, by deleting each half-sib family in turn a reduced G matrix was calculated and the deleted family was added back to the dataset. Thus, *family – 1* pseudovalues for each G matrix were calculated using the observed and the reduced G matrix. Because pseudovalues have the same distribution as G, they can be compared using a MANOVA with ethanol treatment as categorical variable and pseudovalues as dependent variables. The jackknife procedure was performed using a script kindly provided by Derek Roff. Since the inclusion of ‘additional’ effects (i.e., replicated line, sex, respirometric chamber) is relatively complex, we performed main effects ANOVA for each phenotypic variable using those factors that showed significant effects on the variable to obtain residuals, which were used as new dependent variables for pseudovalues estimation (Derek Roff, *personal communication*).

## Results

### Trait means

**Figure 1:**
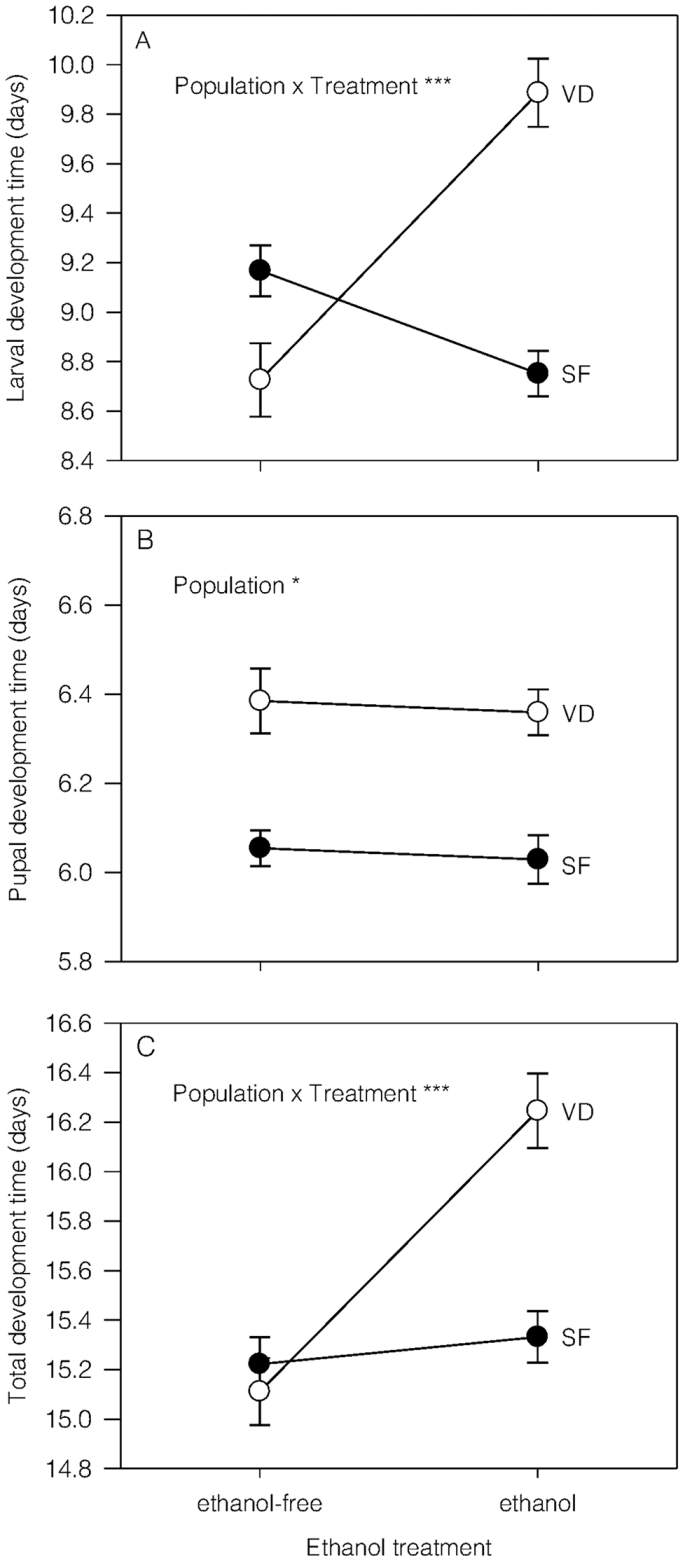
Reaction norms of development traits for *Drosophila melanogaster* flies developed in contrasting ethanol conditions. Average (± SE) larval (A), pupal (B) and total (C) development time for flies (sexes were pooled) from the San Fernando (SF) and the Valdivia (VD) populations reared in ethanol-free (0% ethanol) and ethanol-supplemented (7% ethanol) conditions. Significant effects are expressed as: *P<0.05, **P<0.01, ***P<0.001.

For larval (Fig. 1a) and total (Fig. 1c) development times, we found a significant interaction between population and ethanol treatments (*F*_1,737_ = 47.47, *P*<0.001; *F*_1,741_ = 14.10, *P*<0.001, respectively). Larval and total development times were shorter under ethanol-free than ethanol-supplemented conditions for individual from the Valdivia population (Unequal N HSD: *P*<0.001 for both cases), while development times tended to be similar between ethanol conditions in the San Fernando population (Unequal N HSD: *P* = 0.05 and Unequal N HSD: *P* = 0.79 for larval and total development time). On the other hand, pupal development time (Fig. 1b) was longer for individuals from the Valdivia than the San Fernando populations (*F*_1,4_ = 9.84, *P* = 0.03) but ethanol was not found to have a significant effect, nor was there interaction between main effects (*F*_1,732_ = 0.05, *P* = 0.82 and *F*_1,732_ = 0.76, *P* = 0.38, respectively).

**Figure 2:**
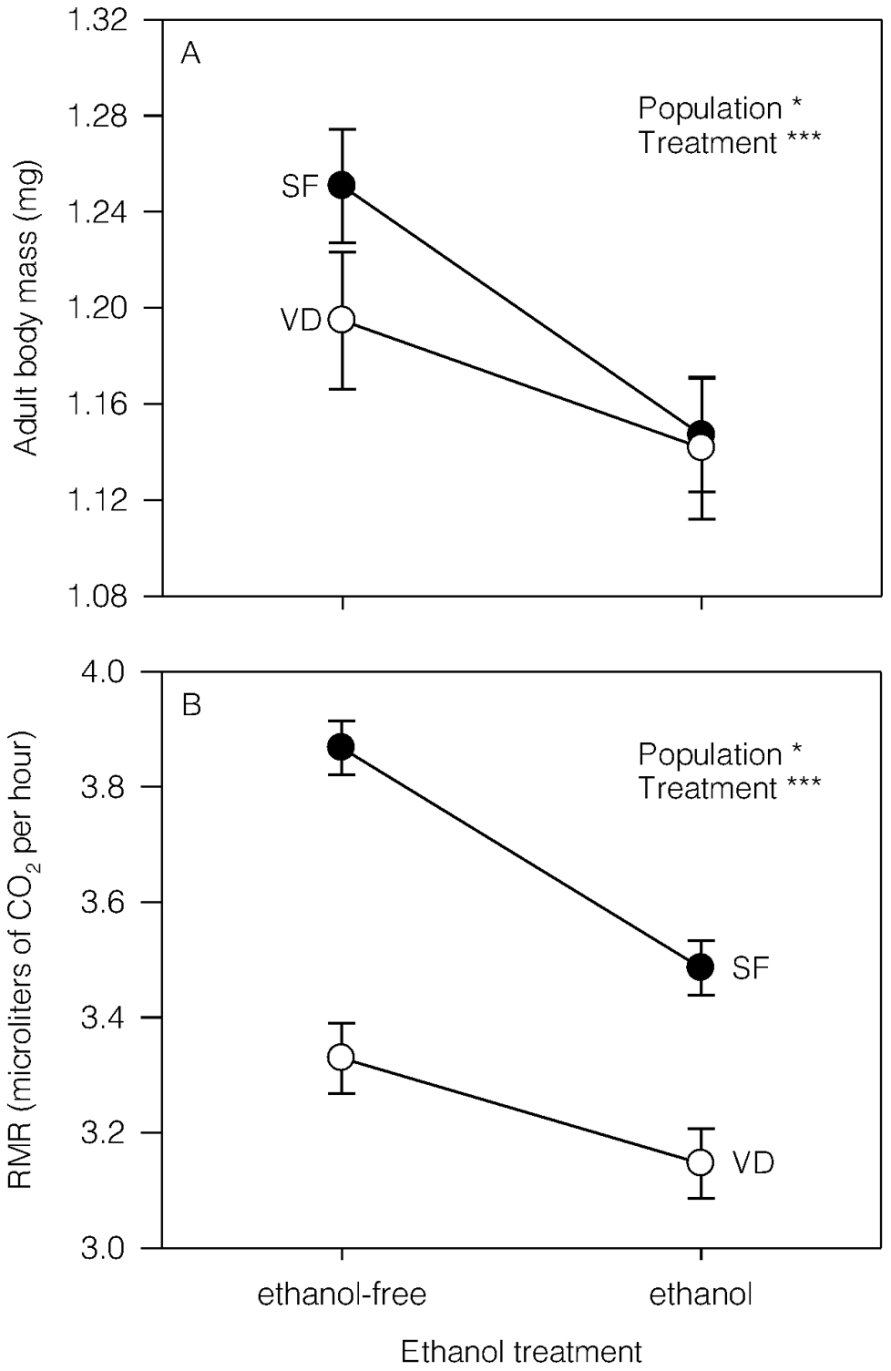
Reaction norms of body mass and metabolic rate for *Drosophila melanogaster* flies developed in contrasting ethanol conditions. Average (± SE) adult body size (A) and routine metabolic rate (RMR; B) for flies (sexes were pooled) from the San Fernando (SF) and the Valdivia (VD) populations reared in ethanol-free (0% ethanol) and ethanol-supplemented (7% ethanol) conditions. Significant effects are expressed as: *P<0.05, **P<0.01, ***P<0.001.

For adult body mass (Fig. 2a), flies from the San Fernando population were larger than those from the Valdivia population (*F*_1,4_ = 12.88, *P* = 0.02), whereas flies from both populations reared in ethanol-free conditions exhibited larger adult body mass than those reared in the ethanol-supplemented treatment (*F*_1,743_ = 30.0, *P*<0.001), but a non-significant interaction was found between these main effects (*F*_1,743_ = 1.70, *P* = 0.19). Furthermore, we found a significant interaction between sex and ethanol treatment (*F*_1,743_ = 12.40, *P*<0.001) because females reared in ethanol-supplemented condition exhibited lower body mass than those reared in ethanol-free conditions, whereas body mass of males did not differ between ethanol treatments. This differential response to ethanol between sexes could have consequences for statistical analysis of RMR because body mass was fit as a common covariate. Thus, slopes between body mass and RMR were not significantly different between sexes (*β*_FEMALE_  = 0.89±0.10 and *β*_MALE_  = 0.94±0.18; *F*_1,749_ = 0.07, *P* = 0.79). We also compared the slopes between body mass and RMR and these were not significantly different between ethanol treatments (*β*_ETHANOL-FREE_  = 0.69 ± 0.05 and *β*_ETHANOL-SUPPLEMENTED_  = 0.60±0.07; *F*_1,749_  = 0.86, *P* = 0.35). These results suggest that there is no statistical conflict in using body mass as a common covariate in analyses including sex and ethanol as main factors. Therefore, RMR analysis (Fig. 2b) found significant differences between populations (*F*_1,4_  = 8.32, *P* = 0.04) and ethanol treatment (*F*_1,726_  = 10.89, *P* = 0.001), but non-significant interaction between them (*F*_1,726_  = 1.84, *P* = 0.18). Flies from the San Fernando population exhibited higher RMR than flies from the Valdivia population (Unequal N HSD: *P*<0.001), whereas flies reared in ethanol-supplemented conditions showed lower RMR than those reared in ethanol-free conditions (Unequal N HSD: *P*<0.001).

### Heritabilities and genetic correlations

Significant heritabilities were found for all traits measured in both populations reared in different ethanol treatments (Table 1; see Tables S3–S6 for comparisons of DIC values between the complete and constrained models), suggesting potential for evolutionary responses in measured traits of *D. melanogaster*. For the San Fernando population, higher heritabilities were found for larval and total development times in ethanol-free than in ethanol-supplemented conditions (*h* ^2^  =  0.64 *vs*. 0.06 and 0.60 *vs*. 0.08, respectively). On the other hand, heritabilities for pupal development time, adult body mass and RMR were remarkably similar between ethanol conditions for the San Fernando population (Table 1). In contrast, heritabilities of most traits for the Valdivia population were relatively low and similar between ethanol treatments (Table 1). Additive genetic, common-environmental, replicated line and non-common environmental or residual variances are shown in Tables S3–S6.

**Table 1:** Heritabilities and genetic correlations of developmental and physiological traits for *Drosophila melanogaster* flies developed in contrasting ethanol conditions.

	San Fernando		Valdivia	
	ethanol-free	ethanol-supplemented	ethanol-free	ethanol-supplemented
*Heritabilities*				
log_10_ LDT	**0.64 [0.25, 0.84]**	**0.06 [0.01, 0.34]**	**0.07 [0.01, 0.39]**	**0.05 [0.01, 0.26]**
log_10_ PDT	**0.08 [0.07, 0.08]**	**0.18 [0.17, 0.18]**	**0.06 [0.06, 0.06]**	**0.07 [0.06, 0.07]**
log_10_ TDT	**0.60 [0.21, 0.82]**	**0.08 [0.01, 0.34]**	**0.07 [0.02, 0.43]**	**0.04 [0.01, 0.31]**
log_10_ Mb	**0.11 [0.02, 0.29]**	**0.07 [0.01, 0.26]**	**0.15 [0.01, 0.32]**	**0.14 [0.02, 0.45]**
log_10_ RMR	**0.11 [0.03, 0.35]**	**0.06 [0.01, 0.32]**	**0.07 [0.01, 0.41]**	**0.06 [0.01, 0.34]**
				
*Genetic correlations*				
log_10_ LDT − log_10_ PDT	0.18 [−0.53, 0.72]	−0.01 [−0.69, 0.62]	−0.45 [−0.76, 0.57]	– 0.33 [– 0.79, 0.57]
log_10_ LDT − log_10_ TDT	**0.95 [0.91, 0.98]**	**0.73 [0.18, 0.94]**	**0.64 [0.04, 0.96]**	**0.77 [0.11, 0.93]**
log_10_ LDT − log_10_ Mb	−0.43 [−0.70, 0.06]	−0.46 [−0.72, 0.32]	0.01 [−0.65, 0.51]	−0.29 [−0.70, 0.45]
log_10_ LDT − log_10_ RMR	−**0.63 [**−**0.88,** −**0.16]**	−0.22 [−0.69, 0.53]	0.03 [−0.74, 0.72]	−0.07 [−0.73, 0.62]
log_10_ PDT − log_10_ TDT	0.37 [−0.33, 0.82]	−0.12 [−0.71, 0.62]	0.17 [−0.64, 0.74]	0.37 [−0.51, 0.83]
log_10_ PDT − log_10_ Mb	0.32 [−0.30, 0.67]	−0.01 [−0.59, 0.50]	0.04 [−0.56, 0.61]	−0.16 [−0.61, 0.66]
log_10_ PDT − log_10_ RMR	0.21 [−0.67, 0.57]	−0.64 [−0.88, 0.11]	0.01 [−0.79, 0.62]	−0.27 [−0.84, 0.58]
log_10_ TDT − log_10_ Mb	−0.27 [−0.61, 0.21]	−0.47 [−0.76, 0.24]	−0.01 [−0.68, 0.49]	0.11 [−0.67, 0.54]
log_10_ TDT − log_10_ RMR	−**0.64 [**−**0.88,** −**0.16]**	−0.02 [−0.71, 0.55]	−0.17 [−0.71, 0.70]	−0.51 [−0.81, 0.50]
log_10_ Mb − log_10_ RMR	0.25 [−0.37, 0.59]	0.10 [−0.47, 0.58]	0.13 [−0.35, 0.76]	0.37 [−0.29, 0.81]

Larval development time (LDT), pupal development time (PDT), total development time (TDT), adult body mass (Mb) and routine metabolic rate (RMR) were measured in flies from two Chilean populations (San Fernando and Valdivia) reared in ethanol-free (0% ethanol) and ethanol-supplemented (7% ethanol) conditions. Values between brackets indicate the confidence intervals at 95% for each genetic parameter and those not overlapping zero values are bolded.

Genetic correlations between developmental traits were non-significant, except between larval and total development times, which exhibited significant correlations in all population-ethanol combinations (range: 0.64–0.95; see Table 1 and Tables S3–S6 for comparisons of DIC values between the complete and constrained models). Interestingly, we found significant and negative genetic correlations between RMR and larval development time (*r_g_*  = −0.63) and between RMR and total development time (*r_g_*  = −0.64), but only for individuals from the San Fernando population developed in ethanol-free conditions, suggesting a significant relationship between development time and maintenance costs. In other words, larvae from the San Fernando population developed in ethanol-free conditions with high metabolic rates (e.g., individuals with accelerated metabolic processes) take a shorter time to emerge as adult flies than larvae with low metabolic rates. However, this genetic correlation was not significant either under ethanol-supplemented condition for the San Fernando population or for the Valdivia population (both ethanol treatments) (Tables 1, S4 and S6). Additive genetic, common-environmental, replicated line of each population and non-common environmental or residual effects covariances are shown in Tables S3–S6.

### G matrices comparisons

Analyses performed using the Jackknife-MANOVA method indicate that the evolutionary response of the San Fernando population could depend on the ethanol conditions on which fruit fly are raised because G matrices differed significantly between ethanol treatments (Wilk's λ  = 0.176, *F*_15,41_  = 12.80, *P*<0.001). On the contrary, non-significant differences between ethanol treatments were found for the Valdivia population (Wilk's λ  = 0.410, *F*_15,16_  = 1.53, *P* = 0.20). Univariate analyses after MANOVA for the San Fernando population indicate that additive genetic variance for larval development time (*F*_1,55_  = 5.57, *P* = 0.02) and total development time (*F*_1,55_ = 6.20, *P* = 0.02) differed between ethanol treatments, being larger under ethanol-free conditions (Tables S4 and S5). Similar differences were found for the additive genetic covariance between larval and total development time (*F*_1,55_ = 6.27, *P* = 0.02) and between pupal and total development time (*F*_1,55_ = 4.08, *P* = 0.04).

## Discussion

In the present work, we found that physiological and developmental traits are sensitive to the environmental ethanol at the phenotypic level, but the studied populations exhibited different responses for development traits. According to genetic parameters, we predicted a positive genetic correlation between metabolic rate and development time and/or a negative genetic correlation between development time and body size for flies exposed to ethanol potentially because energy resources are allocated to detoxification instead of growth. In contrast, in favorable environments (e.g., ethanol-free conditions), we expected a negative genetic correlation between metabolic rate and development time because energy for detoxification now is available for other competing functions and thus individuals with higher metabolic rates would grow faster. Changes in genetic parameters mediated by ethanol were not as widespread as expected. We only found a significant genetic correlation between development time and metabolism in ethanol-free conditions. However, this finding was only found in the San Fernando population, which limits our conclusions.

Dietary ethanol at high concentrations has deleterious effects on organisms [17], [22]. In the present study, we found that two populations respond differently to ethanol treatment because larvae from the Valdivia population reared under ethanol-supplemented conditions exhibited longer larval and total development times, whilst larvae from the San Fernando population showed similar developmental times across environments. Previous evidence suggests that developmental traits are sensitive to ethanol conditions, with increasing times in flies selected for high-ethanol tolerance [24], [45]. Despite the fact that testing the latitudinal variation mediated by ethanol was not a formal goal of the present study, these findings are at least not expected according to ethanol adaptation at latitudinal scales [20] and give a preliminary idea about how ethanol responses of fitness-related traits differ between populations. Furthermore, adult body mass also changed depending on ethanol condition in both populations, exhibiting lower means for flies reared in ethanol-supplemented conditions. Reduced body mass could be a consequence of costs related to ethanol detoxification, which seems to be important for individuals from the Valdivia population that also exhibit the longest development times. However, if detoxification costs are really occurring, they are not inducing the expected increase in metabolic rate as has been reported for flies exposed to ethanol vapor [31]. Indeed, RMR findings suggest that flies exposed to ethanol reduced their energy expenditure.

Significant heritabilities were found in the present study at population and ethanol treatment levels. For instance, heritabilities for adult body mass exhibited a narrow range (*h* ^2^  = 0.09–0.17) that is lower than the median values (*h* ^2^_MEDIAN_  = 0.32) reported by Roff & Mousseau [46]. In addition, routine metabolic rate also exhibited significant heritabilities with similar values between ethanol conditions (*h* ^2^ range  = 0.07–0.15), which were also similar to those reported previously in insects [47], [48]. Significant heritabilities were also found for development traits in concordance with the evolutionary response of development time reported in experimental selection studies [24], [44], but heritabilities were considerably higher in ethanol-free than ethanol-supplemented conditions for the San Fernando population. According to this, Blanckenhorn and Heyland [49] proposed that reduced heritability values under stressful environments could be a consequence of increasing the environmental variance or constraints of the expressed genetic variance. This is in concordance with our findings because additive genetic variance of larval development time in ethanol-free conditions was 14 higher than in ethanol-supplemented conditions, whilst the environmental variance in ethanol-supplemented conditions was 3.5 higher than in ethanol-free conditions for the San Fernando population (Tables S3 and S4).

Regarding the bivariate genetic analyses, the significant genetic correlation between larval and total development times suggests that the development rate in *D. melanogaster* is mainly explained by variation in the length of larval phase instead of that the pupal development time, which has been associated with important fitness-related traits as timing of metamorphosis in amphibians (Tejedo & Reques [50]) and insects (Shafiei et al. [51]). On the other hand, we did not find significant genetic correlations between development time and adult body mass as we expected (see also Blanckenhorn & Heyland [49]), which contrasts with previous studies that have reported that this genetic correlation is not only significant but also sensitive to environmental conditions [52], [53]. Interestingly, we found a significant negative genetic correlation between development time and RMR for flies from the San Fernando population developed in ethanol-free, but not in ethanol-supplemented conditions. In agreement with the additive genetic variances for development time and Blanckenhorn and Heyland' proposal [49], this finding is explained because the additive genetic covariance between larval development time and RMR is about 18 times higher in ethanol-free conditions than in ethanol-supplemented conditions (Tables S3 and S4), but this difference was not statistically supported by *a posteriori* analysis of G matrix comparison. In contrast, the Valdivia population exhibited non-significant genetic correlations between metabolism and development time independently of ethanol treatment, which could be the result of a reduced statistical power to detect significant genetic correlations because the low number of half-sib families analyzed for this population. Finally, changes in additive genetic (co)variances within and between larval and total development times for the San Fernando population are enough to induce significant differences between G matrices estimated in contrasting ethanol conditions, which suggests that ethanol could be modulating the expression of some development traits and thus modifying the genetic architecture of *D. melanogaster*.

In conclusion, despite the fact that in only one of the studied populations had a significant genetic correlation between development time and metabolic rate, this finding allows us to suggest that *D. melanogaster* can reduce its development time by paying a metabolic price (increasing metabolic rate) under ethanol-free conditions. It is plausible that this scenario occurs because detoxification systems are switched-off in benign environments, which would cause an increase of the available energy to be allocated to reduce development time. However, further studies are necessary to evaluate the relationship between levels of ethanol detoxifying enzymes, metabolism and fitness-related traits in a direct approach to estimate the detoxification costs mediated by ethanol consumption. Finally, studies evaluating genetic correlations between metabolic rate and fitness-related traits are scarce in insects, but recently Jumbo-Lucioni et al. [54] reported non-significant genetic correlations between metabolism and competitive fitness in *D. melanogaster*. However, evidence of negative directional selection on metabolism supports the hypothesis that energy saving strategies have an important impact on fitness [34]. Therefore, our results add an interesting finding that suggests that environmental stress can modify the process of energy allocation, which will contribute to the understanding of how physiological mechanisms could constrain or facilitate adaptive evolution in natural populations.

## Supporting Information

Table S1 **Number dams mated with each sire and total sibs derived from matings which were measured for each population (San Fernando and Valdivia, Chile) reared in ethanol-free and ethanol-supplemented conditions.**
(DOC)

Table S2 **Mixed-model ANOVA testing effects of population, ethanol treatment, sex, interactions between these fixed effects, and replicated line nested within population (as random effect) on larval development time, pupal development time, total development time, adult body mass and routine metabolic rate of** ***Drosophila melanogaster*** **.**
(DOC)

Table S3 **Variance and covariance components estimated for additive genetic (** ***A*** **), common-environmental (** ***C*** **), population replicate (** ***R*** **) and non-common environmental (** ***E*** **) effects of measured traits (log_10_-transformed) in** ***Drosophila melanogaster*** **from the San Fernando population (Chile) reared in ethanol-free conditions.** Values of the deviance information criterion (DIC) are provided for the complete model (*ACRE*) and the model excluding the additive genetic component (*CRE*).
(DOC)

Table S4 **Variance and covariance components estimated for additive genetic (** ***A*** **), common-environmental (** ***C*** **), population replicate (** ***R*** **) and non-common environmental (** ***E*** **) effects of measured traits (log_10_-transformed) in** ***Drosophila melanogaster*** **from the San Fernando population (Chile) reared in ethanol-supplemented conditions.** Values of the deviance information criterion (DIC) are provided for the complete model (*ACRE*) and the model excluding the additive genetic component (*CRE*).
(DOC)

Table S5 **Variance and covariance components estimated for additive genetic (** ***A*** **), common-environmental (** ***C*** **), population replicate (** ***R*** **) and non-common environmental (** ***E*** **) effects of measured traits (log_10_-transformed) in** ***Drosophila melanogaster*** **from the Valdivia population (Chile) reared in ethanol-free conditions.** Values of the deviance information criterion (DIC) are provided for the complete model (*ACRE*) and the model excluding the additive genetic component (*CRE*).
(DOC)

Table S6 **Variance and covariance components estimated for additive genetic (** ***A*** **), common-environmental (** ***C*** **), population replicate (** ***R*** **) and non-common environmental (** ***E*** **) effects of measured traits (log_10_-transformed) in** ***Drosophila melanogaster*** **from the Valdivia population (Chile) reared in ethanol-supplemented conditions.** Values of the deviance information criterion (DIC) are provided for the complete model (*ACRE*) and the model excluding the additive genetic component (*CRE*).
(DOC)
