# Supplementary material for: Phenotypic and Genetic Effects of Contrasting Ethanol Environments on Physiological and Developmental Traits in Drosophila melanogaster
Source: PLoS One. 2013 Mar 7;8(3):e58920. doi: 10.1371/journal.pone.0058920 (PMC3591359; doi:10.1371/journal.pone.0058920)
Supplement: Table S1 — Number dams mated with each sire and total sibs derived from matings which were measured for each population (San Fernando and Valdivia, Chile) reared in ethanol-free and ethanol-supplemented conditions. (DOC) [file pone.0058920.s001.doc]

Table S1. Number dams mated with each sire and total sibs derived from matings which were measured for each population (San Fernando and Valdivia, Chile) reared in ethanol-free and ethanol-supplemented conditions.

| San Fernando − Free | | |  | San Fernando − Ethanol | | |  | Valdivia − Free | | |  | Valdivia − Ethanol | | |
| --- | --- | --- | --- | --- | --- | --- | --- | --- | --- | --- | --- | --- | --- | --- |
| Sire | Dams | Sibs |  | Sire | Dams | Sibs |  | Sire | Dams | Sibs |  | Sire | Dams | Sibs |
| 1 | 3 | 11 |  | 1 | 3 | 7 |  | 1 | 3 | 10 |  | 1 | 3 | 7 |
| 2 | 3 | 14 |  | 2 | 3 | 12 |  | 2 | 2 | 7 |  | 2 | 1 | 8 |
| 3 | 3 | 12 |  | 3 | 3 | 11 |  | 3 | 4 | 13 |  | 3 | 3 | 9 |
| 4 | 3 | 12 |  | 4 | 3 | 6 |  | 4 | 2 | 7 |  | 4 | 2 | 6 |
| 5 | 3 | 9 |  | 5 | 3 | 11 |  | 5 | 3 | 10 |  | 5 | 3 | 11 |
| 6 | 3 | 12 |  | 6 | 3 | 7 |  | 6 | 2 | 7 |  | 6 | 2 | 8 |
| 7 | 3 | 16 |  | 7 | 1 | 1 |  | 7 | 2 | 9 |  | 7 | 2 | 8 |
| 8 | 3 | 15 |  | 8 | 3 | 10 |  | 8 | 2 | 8 |  | 8 | 1 | 3 |
| 9 | 3 | 13 |  | 9 | 2 | 3 |  | 9 | 2 | 9 |  | 9 | 3 | 7 |
| 10 | 3 | 15 |  | 10 | 3 | 8 |  | 10 | 2 | 7 |  | 10 | 3 | 7 |
| 11 | 1 | 4 |  | 11 | 0 | 0 |  | 11 | 3 | 10 |  | 11 | 3 | 7 |
| 12 | 3 | 13 |  | 12 | 2 | 7 |  | 12 | 2 | 9 |  | 12 | 2 | 4 |
| 13 | 1 | 4 |  | 13 | 1 | 4 |  | 13 | 2 | 5 |  | 13 | 2 | 5 |
| 14 | 3 | 16 |  | 14 | 2 | 5 |  | 14 | 3 | 8 |  | 14 | 2 | 5 |
| 15 | 1 | 4 |  | 15 | 1 | 2 |  | 15 | 2 | 5 |  | 15 | 2 | 8 |
| 16 | 2 | 9 |  | 16 | 1 | 5 |  | 16 | 3 | 11 |  | 16 | 3 | 11 |
| 17 | 2 | 7 |  | 17 | 2 | 7 |  |  |  |  |  |  |  |  |
| 18 | 3 | 11 |  | 18 | 3 | 10 |  |  |  |  |  |  |  |  |
| 19 | 3 | 10 |  | 19 | 3 | 10 |  |  |  |  |  |  |  |  |
| 20 | 4 | 10 |  | 20 | 3 | 9 |  |  |  |  |  |  |  |  |
| 21 | 2 | 9 |  | 21 | 3 | 9 |  |  |  |  |  |  |  |  |
| 22 | 3 | 10 |  | 22 | 3 | 12 |  |  |  |  |  |  |  |  |
| 23 | 4 | 10 |  | 23 | 3 | 7 |  |  |  |  |  |  |  |  |
| 24 | 1 | 4 |  | 24 | 1 | 6 |  |  |  |  |  |  |  |  |
| 25 | 2 | 8 |  | 25 | 2 | 6 |  |  |  |  |  |  |  |  |
| 26 | 2 | 8 |  | 26 | 3 | 9 |  |  |  |  |  |  |  |  |
| 27 | 1 | 4 |  | 27 | 1 | 5 |  |  |  |  |  |  |  |  |
| 28 | 4 | 17 |  | 28 | 4 | 12 |  |  |  |  |  |  |  |  |
| 29 | 2 | 6 |  | 29 | 2 | 4 |  |  |  |  |  |  |  |  |
|  |  |  |  |  |  |  |  |  |  |  |  |  |  |  |
|  |  |  |  |  |  |  |  |  |  |  |  |  |  |  |
