# Supplementary material for: Phenotypic and Genetic Effects of Contrasting Ethanol Environments on Physiological and Developmental Traits in Drosophila melanogaster
Source: PLoS One. 2013 Mar 7;8(3):e58920. doi: 10.1371/journal.pone.0058920 (PMC3591359; doi:10.1371/journal.pone.0058920)
Supplement: Table S2 — Mixed-model ANOVA testing effects of population, ethanol treatment, sex, interactions between these fixed effects, and replicated line nested within population (as random effect) on larval development time, pupal development time, total development time, adult body mass and routine metabolic rate of Drosophila melanogaster . (DOC) [file pone.0058920.s002.doc]

Table S2. Mixed-model ANOVA testing effects of population, ethanol treatment, sex, interactions between these fixed effects, and replicated line nested within population (as random effect) on larval development time, pupal development time, total development time, adult body mass and routine metabolic rate of *Drosophila melanogaster*.

| Effects | DF_num_ | DF_den_ | MS | F | p |
| --- | --- | --- | --- | --- | --- |
|  |  |  |  |  |  |
| *Log_10_ larval development time* | |  |  |  |  |
| Population | 1 | 4 | 0.0509 | 2.11 | 0.2200 |
| Treatment | 1 | 737 | 0.0663 | 13.53 | 0.0003 |
| Sex | 1 | 737 | 0.0110 | 2.24 | 0.1349 |
| Population × Treatment | 1 | 737 | 0.2326 | 47.47 | < 0.0001 |
| Population × Sex | 1 | 737 | 0.0025 | 0.51 | 0.4754 |
| Treatment × Sex | 1 | 737 | 0.0002 | 0.04 | 0.8415 |
| Population × Treatment × Sex | 1 | 737 | 0.0001 | 0.02 | 0.8876 |
| Replicate(Population) | 4 | 737 | 0.0241 | 4.92 | 0.0006 |
| Error | 737 |  | 0.0049 |  |  |
|  |  |  |  |  |  |
| *Log_10_ pupal development time* | |  |  |  |  |
| Population | 1 | 4 | 0.0787 | 9.84 | 0.0350 |
| Treatment | 1 | 732 | 0.0001 | 0.05 | 0.8231 |
| Sex | 1 | 732 | 0.0411 | 19.57 | < 0.0001 |
| Population × Treatment | 1 | 732 | 0.0016 | 0.76 | 0.3836 |
| Population × Sex | 1 | 732 | 0.0008 | 0.38 | 0.5378 |
| Treatment × Sex | 1 | 732 | 0.0016 | 0.76 | 0.3836 |
| Population × Treatment × Sex | 1 | 732 | 0.0039 | 1.86 | 0.1730 |
| Replicate(Population) | 4 | 732 | 0.0080 | 3.81 | 0.0045 |
| Error | 732 |  | 0.0021 |  |  |
|  |  |  |  |  |  |
| *Log_10_ total development time* |  |  |  |  |  |
| Population | 1 | 4 | 0.0306 | 2.76 | 0.1720 |
| Treatment | 1 | 741 | 0.0485 | 21.25 | < 0.0001 |
| Sex | 1 | 741 | 0.0010 | 0.50 | 0.4797 |
| Population × Treatment | 1 | 741 | 0.0282 | 14.10 | 0.0002 |
| Population × Sex | 1 | 741 | 0.0006 | 0.30 | 0.5840 |
| Treatment × Sex | 1 | 741 | 0.0001 | 0.05 | 0.8231 |
| Population × Treatment × Sex | 1 | 741 | 0.0006 | 0.30 | 0.5840 |
| Replicate(Population) | 4 | 741 | 0.0111 | 5.55 | 0.0002 |
| Error | 741 |  | 0.0020 |  |  |
|  |  |  |  |  |  |
| *Log_10_ adult body mass* |  |  |  |  |  |
| Population | 1 | 4 | 0.0103 | 12.88 | 0.0230 |
| Treatment | 1 | 743 | 0.0300 | 30.00 | < 0.0001 |
| Sex | 1 | 743 | 2.4425 | 2444.5 | < 0.0001 |
| Population × Treatment | 1 | 743 | 0.0017 | 1.70 | 0.1927 |
| Population × Sex | 1 | 743 | 0.0119 | 11.90 | 0.0005 |
| Treatment × Sex | 1 | 743 | 0.0124 | 12.40 | 0.0005 |
| Population × Treatment × Sex | 1 | 743 | 0.0013 | 1.30 | 0.2546 |
| Replicate(Population) | 4 | 743 | 0.0008 | 0.80 | 0.5253 |
| Error | 743 |  | 0.0010 |  |  |
|  |  |  |  |  |  |
| *Log_10_ routine metabolic rate* |  |  |  |  |  |
| Population | 1 | 4 | 0.2788 | 8.3224 | 0.0448 |
| Treatment | 1 | 726 | 0.0610 | 10.993 | 0.0010 |
| Sex | 1 | 726 | 0.0029 | 0.5179 | 0.4720 |
| Population × Treatment | 1 | 726 | 0.0103 | 1.8393 | 0.1755 |
| Population × Sex | 1 | 726 | 0.0315 | 5.6250 | 0.0180 |
| Treatment × Sex | 1 | 726 | 0.0064 | 1.1429 | 0.2854 |
| Population × Treatment × Sex | 1 | 726 | 0.0003 | 0.0536 | 0.8170 |
| Replicate(Population) | 4 | 726 | 0.0335 | 5.9821 | 0.0001 |
| Metabolic chamber | 6 | 726 | 0.0235 | 4.1964 | 0.0004 |
| Record time | 8 | 726 | 0.0169 | 3.0179 | 0.0024 |
| Log_10_ adult body mass | 1 | 726 | 0.3765 | 67.232 | < 0.0001 |
| Error | 726 |  | 0.0056 |  |  |
