# Supplementary material for: Phenotypic and Genetic Effects of Contrasting Ethanol Environments on Physiological and Developmental Traits in Drosophila melanogaster
Source: PLoS One. 2013 Mar 7;8(3):e58920. doi: 10.1371/journal.pone.0058920 (PMC3591359; doi:10.1371/journal.pone.0058920)
Supplement: Table S3 — Variance and covariance components estimated for additive genetic ( A ), common-environmental ( C ), population replicate ( R ) and non-common environmental ( E ) effects of measured traits (log10-transformed) in Drosophila melanogaster from the San Fernando population (Chile) reared in ethanol-free conditions. Values of the deviance information criterion (DIC) are provided for the complete model (ACRE) and the model excluding the additive genetic component (CRE). (DOC) [file pone.0058920.s003.doc]

Table S3. Variance and covariance components estimated for additive genetic (*A*), common-environmental (*C*), population replicate (*R*) and non-common environmental (*E*) effects of measured traits (log_10_-transformed) in *Drosophila melanogaster* from the San Fernando population (Chile) reared in ethanol-free conditions. Values of the deviance information criterion (DIC) are provided for the complete model (*ACRE*) and the model excluding the additive genetic component (*CRE*).

|  | Random effects | | | |  | DIC | |
| --- | --- | --- | --- | --- | --- | --- | --- |
|  | *A* | *C* | *R* | *E* |  | *ACRE* | *CRE* |
| *Variance* |  |  |  |  |  |  |  |
| Larval development time (LDT) | 0.00423 | 0.00040 | 0.00073 | 0.00063 |  | – 1103.86 | – 839.13 |
| Pupal development time (PDT) | 0.00018 | 0.00015 | 0.00041 | 0.00163 |  | – 984.10 | – 975.78 |
| Total development time (TDT) | 0.00174 | 0.00014 | 0.00026 | 0.00035 |  | – 1328.75 | – 1078.02 |
| Adult body mass (Mb) | 0.00075 | 0.00061 | 0.00250 | 0.00233 |  | – 866.66 | – 851.80 |
| Routine metabolic rate (RMR) | 0.00101 | 0.00047 | 0.00132 | 0.00425 |  | – 699.58 | – 682.58 |
| *Covariance* |  |  |  |  |  |  |  |
| LDT − PDT | 0.00016 | 0.00002 | 0.00008 | – 0.00058 |  | – 2076.04 | – 2047.72 |
| LDT − TDT | 0.00247 | 0.00015 | 0.00018 | 0.00033 |  | – 2700.12 | – 2391.81 |
| LDT − Mb | – 0.00062 | – 0.00003 | 0.00060 | – 0.00025 |  | – 1955.14 | – 1921.25 |
| LDT − RMR | – 0.00125 | – 0.00015 | 0.00087 | – 0.00004 |  | – 1816.23 | – 1750.88 |
| PDT − TDT | 0.00018 | 0.00017 | – 0.00012 | 0.00042 |  | – 2320.11 | – 2305.09 |
| PDT − Mb | 0.00012 | 0.00006 | – 0.00025 | 0.00007 |  | – 1863.65 | – 1855.53 |
| PDT − RMR | 0.00010 | 0.00001 | – 0.00005 | – 0.00002 |  | – 1878.17 | – 1673.52 |
| TDT − Mb | – 0.00026 | 0.000001 | 0.00020 | – 0.00016 |  | – 2178.27 | – 2153.42 |
| TDT − RMR | – 0.00082 | – 0.00002 | 0.000001 | – 0.00027 |  | – 2044.71 | – 1979.89 |
| Mb − RMR | 0.00012 | 0.00007 | 0.00115 | 0.00097 |  | – 1564.23 | – 1560.93 |
