# Supplementary material for: Phenotypic and Genetic Effects of Contrasting Ethanol Environments on Physiological and Developmental Traits in Drosophila melanogaster
Source: PLoS One. 2013 Mar 7;8(3):e58920. doi: 10.1371/journal.pone.0058920 (PMC3591359; doi:10.1371/journal.pone.0058920)
Supplement: Table S4 — Variance and covariance components estimated for additive genetic ( A ), common-environmental ( C ), population replicate ( R ) and non-common environmental ( E ) effects of measured traits (log10-transformed) in Drosophila melanogaster from the San Fernando population (Chile) reared in ethanol-supplemented conditions. Values of the deviance information criterion (DIC) are provided for the complete model (ACRE) and the model excluding the additive genetic component (CRE). (DOC) [file pone.0058920.s004.doc]

Table S4. Variance and covariance components estimated for additive genetic (*A*), common-environmental (*C*), population replicate (*R*) and non-common environmental (*E*) effects of measured traits (log_10_-transformed) in *Drosophila melanogaster* from the San Fernando population (Chile) reared in ethanol-supplemented conditions. Values of the deviance information criterion (DIC) are provided for the complete model (*ACRE*) and the model excluding the additive genetic component (*CRE*).

|  | Random effects | | | |  | DIC | |
| --- | --- | --- | --- | --- | --- | --- | --- |
|  | *A* | *C* | *R* | *E* |  | *ACRE* | *CRE* |
| *Variance* |  |  |  |  |  |  |  |
| Larval development time (LDT) | 0.00030 | 0.00036 | 0.00027 | 0.00275 |  | – 588.16 | – 566.52 |
| Pupal development time (PDT) | 0.00071 | 0.00031 | 0.00049 | 0.00185 |  | – 653.85 | – 628.88 |
| Total development time (TDT) | 0.00015 | 0.00021 | 0.00016 | 0.00112 |  | – 767.98 | – 751.37 |
| Adult body mass (Mb) | 0.00067 | 0.00091 | 0.00160 | 0.00241 |  | – 599.07 | – 585.68 |
| Routine metabolic rate (RMR) | 0.00050 | 0.00167 | 0.00072 | 0.00242 |  | – 604.79 | – 547.43 |
| *Covariance* |  |  |  |  |  |  |  |
| LDT − PDT | 0.00001 | 0.00012 | – 0.00013 | – 0.00019 |  | – 1261.23 | – 1242.09 |
| LDT − TDT | 0.00014 | 0.00023 | 0.00012 | 0.00151 |  | – 1625.54 | – 1616.52 |
| LDT − Mb | – 0.00011 | – 0.00032 | – 0.00083 | – 0.00053 |  | – 1204.92 | – 1193.11 |
| LDT − RMR | – 0.00007 | 0.00004 | – 0.00007 | – 0.00039 |  | – 1237.26 | – 1226.07 |
| PDT − TDT | – 0.00006 | 0.00006 | – 0.00007 | 0.00068 |  | – 1492.64 | – 1480.00 |
| PDT − Mb | 0.00001 | – 0.00001 | 0.00026 | – 0.00016 |  | – 1270.18 | – 1262.31 |
| PDT − RMR | – 0.00040 | – 0.00035 | – 0.00005 | 0.00053 |  | – 1277.89 | – 1221.66 |
| TDT − Mb | – 0.00013 | – 0.00016 | – 0.00012 | – 0.00036 |  | – 1396.78 | – 1382.26 |
| TDT − RMR | – 0.00001 | – 0.00015 | 0.00018 | – 0.00018 |  | – 1415.85 | – 1401.03 |
| Mb − RMR | 0.00010 | 0.00022 | 0.00017 | 0.00086 |  | – 1213.25 | – 1204.50 |
