# Supplementary material for: Phenotypic and Genetic Effects of Contrasting Ethanol Environments on Physiological and Developmental Traits in Drosophila melanogaster
Source: PLoS One. 2013 Mar 7;8(3):e58920. doi: 10.1371/journal.pone.0058920 (PMC3591359; doi:10.1371/journal.pone.0058920)
Supplement: Table S5 — Variance and covariance components estimated for additive genetic ( A ), common-environmental ( C ), population replicate ( R ) and non-common environmental ( E ) effects of measured traits (log10-transformed) in Drosophila melanogaster from the Valdivia population (Chile) reared in ethanol-free conditions. Values of the deviance information criterion (DIC) are provided for the complete model (ACRE) and the model excluding the additive genetic component (CRE). (DOC) [file pone.0058920.s005.doc]

Table S5. Variance and covariance components estimated for additive genetic (*A*), common-environmental (*C*), population replicate (*R*) and non-common environmental (*E*) effects of measured traits (log_10_-transformed) in *Drosophila melanogaster* from the Valdivia population (Chile) reared in ethanol-free conditions. Values of the deviance information criterion (DIC) are provided for the complete model (*ACRE*) and the model excluding the additive genetic component (*CRE*).

|  | Random effects | | | |  | DIC | |
| --- | --- | --- | --- | --- | --- | --- | --- |
|  | *A* | *C* | *R* | *E* |  | *ACRE* | *CRE* |
| *Variance* |  |  |  |  |  |  |  |
| Larval development time (LDT) | 0.00057 | 0.00045 | 0.00077 | 0.00464 |  | – 306.42 | – 293.98 |
| Pupal development time (PDT) | 0.00018 | 0.00019 | 0.00023 | 0.00220 |  | – 415.84 | – 412.47 |
| Total development time (TDT) | 0.00021 | 0.00018 | 0.00022 | 0.00136 |  | – 485.14 | – 472.62 |
| Adult body mass (Mb) | 0.00064 | 0.00045 | 0.00111 | 0.00215 |  | – 417.53 | – 410.09 |
| Routine metabolic rate (RMR) | 0.00054 | 0.00051 | 0.00051 | 0.00436 |  | – 307.04 | – 299.46 |
| *Covariance* |  |  |  |  |  |  |  |
| LDT − PDT | – 0.00002 | 0.00003 | – 0.00033 | – 0.00189 |  | – 785.63 | – 780.19 |
| LDT − TDT | 0.00017 | 0.00013 | 0.00008 | 0.00228 |  | – 952.37 | – 947.68 |
| LDT − Mb | 0.00001 | 0.00005 | – 0.00009 | – 0.00067 |  | – 738.04 | – 732.44 |
| LDT − RMR | 0.00003 | – 0.00017 | – 0.00014 | – 0.00011 |  | – 614.36 | – 601.66 |
| PDT − TDT | – 0.00003 | 0.00009 | – 0.00005 | 0.00010 |  | – 914.70 | – 904.00 |
| PDT − Mb | 0.00002 | – 0.00006 | – 0.00003 | – 0.00015 |  | – 839.66 | – 835.56 |
| PDT − RMR | 0.00001 | 0.00003 | 0.00001 | – 0.00079 |  | – 725.37 | – 718.69 |
| TDT − Mb | – 0.00005 | 0.00002 | – 0.00002 | – 0.00035 |  | – 922.88 | – 917.60 |
| TDT − RMR | 0.00005 | – 0.00005 | 0.00002 | – 0.00036 |  | – 803.90 | – 790.70 |
| Mb − RMR | 0.00010 | 0.00012 | – 0.00019 | 0.00096 |  | – 730.69 | – 725.13 |
