# Supplementary material for: Phenotypic and Genetic Effects of Contrasting Ethanol Environments on Physiological and Developmental Traits in Drosophila melanogaster
Source: PLoS One. 2013 Mar 7;8(3):e58920. doi: 10.1371/journal.pone.0058920 (PMC3591359; doi:10.1371/journal.pone.0058920)
Supplement: Table S6 — Variance and covariance components estimated for additive genetic ( A ), common-environmental ( C ), population replicate ( R ) and non-common environmental ( E ) effects of measured traits (log10-transformed) in Drosophila melanogaster from the Valdivia population (Chile) reared in ethanol-supplemented conditions. Values of the deviance information criterion (DIC) are provided for the complete model (ACRE) and the model excluding the additive genetic component (CRE). (DOC) [file pone.0058920.s006.doc]

Table S6. Variance and covariance components estimated for additive genetic (*A*), common-environmental (*C*), population replicate (*R*) and non-common environmental (*E*) effects of measured traits (log_10_-transformed) in *Drosophila melanogaster* from the Valdivia population (Chile) reared in ethanol-supplemented conditions. Values of the deviance information criterion (DIC) are provided for the complete model (*ACRE*) and the model excluding the additive genetic component (*CRE*).

|  | Random effects | | | |  | DIC | |
| --- | --- | --- | --- | --- | --- | --- | --- |
|  | *A* | *C* | *R* | *E* |  | *ACRE* | *CRE* |
| *Variance* |  |  |  |  |  |  |  |
| Larval development time (LDT) | 0.00033 | 0.00242 | 0.00050 | 0.00112 |  | – 467.79 | – 414.81 |
| Pupal development time (PDT) | 0.00012 | 0.00008 | 0.00015 | 0.00111 |  | – 418.76 | – 415.82 |
| Total development time (TDT) | 0.00009 | 0.00099 | 0.00015 | 0.00042 |  | – 549.04 | – 495.43 |
| Adult body mass (Mb) | 0.00119 | 0.00121 | 0.00238 | 0.00251 |  | – 334.39 | – 309.81 |
| Routine metabolic rate (RMR) | 0.00066 | 0.00057 | 0.00059 | 0.00449 |  | – 277.34 | – 268.13 |
| *Covariance* |  |  |  |  |  |  |  |
| LDT − PDT | – 0.00006 | 0.00012 | 0.00012 | 0.00002 |  | – 895.00 | – 866.00 |
| LDT − TDT | 0.00011 | 0.00142 | 0.00026 | 0.00060 |  | – 1116.73 | – 1064.48 |
| LDT − Mb | – 0.00011 | 0.00021 | 0.00009 | – 0.00030 |  | – 808.82 | – 801.95 |
| LDT − RMR | 0.00010 | 0.00006 | – 0.00048 | 0.00013 |  | – 753.21 | – 745.16 |
| PDT − TDT | 0.00001 | 0.00016 | 0.000001 | 0.00042 |  | – 1005.79 | – 998.77 |
| PDT − Mb | – 0.00005 | – 0.00015 | – 0.00009 | 0.00039 |  | – 773.77 | – 766.68 |
| PDT − RMR | – 0.00002 | – 0.00001 | 0.00001 | – 0.00037 |  | – 700.79 | – 689.53 |
| TDT − Mb | 0.00005 | 0.00006 | – 0.00015 | 0.00011 |  | – 886.74 | – 883.51 |
| TDT − RMR | – 0.00013 | 0.00001 | – 0.00011 | – 0.00020 |  | – 826.34 | – 816.06 |
| Mb − RMR | 0.00043 | 0.00017 | – 0.00014 | 0.00104 |  | – 621.74 | – 609.28 |
